# Transcutaneous electrical acustimulation promotes wound healing in mice by modulating signaling molecules and mitochondria function

**DOI:** 10.1007/s00403-024-03754-y

**Published:** 2025-02-08

**Authors:** Rong Han, Menghua Chen, Wang Peng, Jianbo Yue, Jinlian Hu

**Affiliations:** 1 Department of Biomedical Engineering (BME), City University of Hong Kong, 83 Tat Chee Avenue, Kowloon Tong, Hong Kong, China; 2 Department of Biomedical Science, City University of Hong Kong, 83 Tat Chee Avenue, Kowloon Tong, Hong Kong, China; 3 Aussway Chinese Medicine Centre, 173 East Boundary Road, Bentleigh East, VIC 3165 Australia; 4 Division of Natural and Applied Sciences, Duke Kunshan University, Suzhou, China

**Keywords:** Transcutaneous electrical acustimulation, Wound healing, Signaling molecules, Repair, Mitochondrial dynamics

## Abstract

Previous research has identified a variety of factors that contribute to the development and maintenance of wounds. Concurrently, electroacupuncture has been demonstrated to facilitate wound healing. However, the effects of transcutaneous electrical acustimulation (TEA) on wound healing, as well as its relationship with key factors such as Wnt3a, TGF-β, Akt, c-Myc, VEGF-A, SP1, nitric oxide (NO), and mitochondrial function, remain largely unexplored. We hypothesize that TEA will activate the signaling factors and enhance mitochondrial functions to promote the repair of skin wounds in mice. An in vivo experimental study was conducted utilizing mouse models with skin wounds. The study comprised three groups: a TEA treatment with wound group, a skin wound model group, and a control group. Wound areas were measured by calculating the product of the length and width of each wound using calipers. Single-cell suspensions were prepared by excising the wound and the immediately surrounding tissue. These suspensions were stained with Trypan blue to assess cell viability, with specific probes to measure the rate of reactive oxygen species (ROS) positivity, and with reagents to quantify NO content. Western blotting (WB) was employed to evaluate protein levels associated with tissue changes, while quantitative polymerase chain reaction (qPCR) was used to assess RNA expression levels. Immunofluorescence staining was performed to visualize protein content and other relevant cellular structures within tissue sections. TEA exhibited anti-inflammatory properties and promoted wound healing in mice. Western blot analysis revealed that TEA enhanced the expression of proteins associated with Wnt3a, TGF-β, Akt, c-Myc, VEGF-A, and SP1 during the wound healing process. Immunofluorescence staining of tissue sections indicated that TEA upregulated the expression of COL1A1, MFN1, GRP75, GRP78, GRP75/ROS, GRP78/ROS, ISCU, and UCP1 while downregulating FIS1. Additionally, qPCR results demonstrated that TEA promoted the expression of IL-10 and miRNA205-5p while inhibiting MMP9 levels. TEA modulates various signaling molecules, influences chaperone proteins related to stress recovery responses, along with mitochondrial dynamics and metabolism.

**Graphical abstract:**

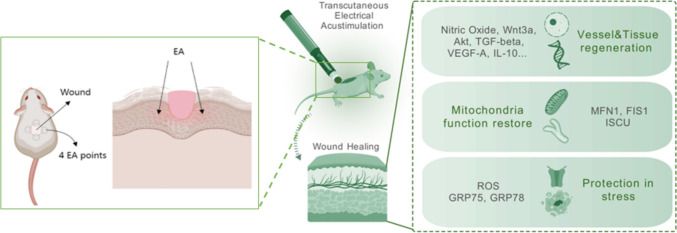

## Background

Wound healing is a complex process regulated by various cells, cytokines, and growth factors [1]. Many cell types with different roles in hemostasis, inflammation, proliferation, re-epithelialization, and remodeling are synchronized both spatially and temporally in this process [1]. The role of several signaling factors in wound healing, including TGF-β (Transforming growth factor beta), Wnt (Wingless/Integrated), Akt (Protein Kinase B, PKB), SP1 (Specificity Protein 1), c-Myc (Cellular Myelocytomatosis Viral Oncogene Homolog),, VEGF-A (Vascular Endothelial Growth Factor A), and NO (Nitric Oxide), is critical to the wound healing process [2–11]. For instance, the TGF-β signaling pathway is important for granulation tissue development, angiogenesis, inflammation, and re-epithelialization during wound healing [2]. Furthermore, by targeting c-Myc and Hes1, Wnt and Notch signaling pathways have been demonstrated to control the migration, proliferation, and differentiation of cells, hence being important for skin tissue repair [3]. Nitric oxide (NO) is mainly synthesized by macrophages in the wound and can stimulate re-epithelialization, ang it acts as a signaling molecule and can activate the PI3K/Akt pathway, leading to increased keratinocyte migration and proliferation [4]. In the skin, the PI3K/Akt pathway controls cell migration, differentiation, and proliferation [5]. Furthermore, a transcription factor called c-Myc is crucial for cell division [3, 6], while VEGF-A and SP1 are known to be involved in promoting cell proliferation and angiogenesis [7, 8].

COL1A1 is a primary component of collagen in the extracellular matrix, essential for providing structural support and facilitating tissue repair during the wound healing process [9]. ISCU is essential for the assembly of iron-sulfur clusters, which are critical for the proper functioning of mitochondrial enzymes involved in energy production [10–12]. Enhanced mitochondrial activity meets the high energy demands of proliferating cells during wound healing [13]. MFN1 is involved in mitochondrial fusion, while FIS1 is crucial for mitochondrial fission [14]. Reverse mitochondria fragmentation can help to recover oxidative stress [15]. UCP1 facilitates thermogenesis in brown adipose tissue and modulates inflammation during wound healing by promoting macrophage activation and energy expenditure [16]. GRP75 assists in protein folding within mitochondria and is involved in the stress response [17]; GRP78 is a chaperone protein that plays a critical role in the endoplasmic reticulum stress response [18]; It aids in cell survival and function during the inflammatory phase of wound healing [18].

Acupuncture, being stimulated on acupoint, can regulate body’s physiological functions, and is used for pain relief, sedation, promotion of blood circulation, and adjustment of muscle tone [19]. Transcutaneous electrical acustimulation (TEA) is a non-invasive emerging tool developed from traditional electroacupuncture therapy [20–22]. There’s also a lot of research on electrical stimulation for wound healing [23–25]. Previous studies have demonstrated the effectiveness of traditional electroacupuncture in promoting wound healing [26–28]. However, they were involved in the invasive nature of traditional electroacupuncture methods [26]. Meanwhile, Intricate signaling pathways and factors underscore their importance in the wound healing process [2–18]. Understanding the specific targets and mechanisms of non-invasive electrical acustimulation in the context of these factors is crucial for developing effective therapeutic strategies for wound healing [2–18]. The study intends to investigate transcutaneous electrical acustimulation’s wound healing effect, anti-inflammatory properties, and its effects on regulating signaling factors, mitochondria dynamics, metabolism and stress recovery response.

## Methods/materials

### Animal

A total of 18 male Balb/c mice weighing 22–24 g were used in this study. While confined 4 mice in each cage, they enjoyed a 12 h light/dark cycle, at a controlled temperature (37 Celsius degree), and free access to food and drink. Animal experiment ethic has been approved with the numbers of AN-STA-00000095 by animal ethic committee of City University of Hong Kong.

### Model establishment and experimental groups

According to Du 2020 [26], a full-thickness skin resection wound mice model was created. In details, the mice were typically raised for one week, fasted for twelve hours prior to modeling, given xylazine and ketamine injections (100/10 mg/kg) for anesthesia, fixed at five different locations, had the modeling area sheared, had hair removal treatments, saline flushing, iodophor disinfection, and ethanol deiodination. Wound areas are measured by using the product of length and width of the wound using calipers [26]. The iodophor treatment was provided to both the Wound group and the Wound + TEA group to avoid infection. Control group mice undergo the same shaving procedure as the other 2 groups. A punch was used to create a wound model with a diameter of around 1 cm. According to Du et al. [26], rulers are used to determine a wound’s long and short diameters, and the wound area is calculated by multiplying the long diameter by the short diameter.

The mice were randomly divided into three groups with 6 mice in each group: (1) Control, (2) Wound, and (3) Wound + TEA stimulation. The control group mice were conducted no wound establishment and had no TEA intervention but for reference only. Wound group mice were only conducted wound and not receive TEA intervention. Wound + TEA group mice received TEA intervention while having wound. TEA was conducted on mice wound surrounding areas. After 7 days of TEA treatment, mice tissues were collected by use of a puncher on wound surrounding area for quantitative polymerase chain reaction (qPCR), immunofluorescence imaging of tissue section, western blotting (WB), nitrate oxide (NO) detection assay, reactive oxygen species (ROS) detection assay and single-cell suspension solution fabrication. Tissues from the control group mice also collected on day 7. Detailed experiment methods and timelines were described in the following sections and depicted in graphical abstract.

### EA treatment

According to Du 2020 [26], we also conducted TEA on the 4 points around wound. One hand held the mouse at the midpoint of the tail, and the other hand of the same operator held the electroacupuncture pen to perform transcutaneous electrical acustimulation at the acupoints. TEA was then performed using a non-invasive electric acustimulation device. For a total of six minutes per day, four spots surrounding wound areas with each treated 1.5 min at a working voltage in 3.7 V. To avoid wound infection, iodophor and regular diet were provided to the mice. A schematic of TEA was presented in graphical abstract.

### Western blotting

To analyze the effects of TEA on specific proteins, western blotting was performed. The samples of mice tissue were stored in a − 80° freezer until they were manually homogenized with RIPA lysis buffer by use of homogenizer. RIPA lysis buffer was added (Beyotime, Inc.), which contains 1% Triton X-100, protease inhibitors PMSF (Beyotime, Inc.), sodium fluoride, leupeptin, 0.1% sodium dodecyl sulfate (SDS), 150 mM NaCl, sodium orthovanadate, 1% sodium deoxycholate, and ethylenediaminetetraacetic acid (EDTA). After placed for 20 min on ice, the homogenate was centrifuged for 15 min at 4 °C at 15,000 relative centrifugal force (RCF), and the supernatant was collected. The sample buffer was added to above extracted protein solution according to the instructions (Beyotime, Inc) and boiled in a metal bath at 95 °C for 10 min, then stored in a refrigerator at − 80 °C until use. Protein samples were separated by electrophoresis in a 10% SDS-PAGE gel (Beyotime, Inc.) and electrophoretically transferred to polyvinylidene difluoride (PVDF) membranes (Beyotime, Inc.) afterwards. After that, the membranes were blocked at room temperature for an hour with 5% low-fat milk in Tris-buffered saline with 0.1% Tween (TBST) (Beyotime, Inc.). We employed rabbit anti-mouse VEGF-A (1:1000, ABclonal, Inc.) primary antibody, rabbit anti-mouse SP1 (1:1000, ABclonal, Inc.) primary antibody, rabbit anti-mouse c-Myc (1:1000, ABclonal, Inc.) primary antibody, rabbit anti-mouse Wnt3a (1:1000, ABclonal, Inc.) primary antibody, rabbit anti-mouse Akt (1:1000, ABclonal, Inc.) primary antibody, rabbit anti-mouse TGF-Beta (1:1000, ABclonal, Inc.) primary antibody, and goat anti-rabbit (1:10,000, ABclonal, Inc.) secondary antibody. Rabbit anti-mouse GAPDH (1:10,000, ABclonal, Inc.) was employed as an internal control. The signals were recorded using Bio-Rad Chemidoc MP imaging system by use of an ultra-enhanced chemiluminescence (ECL) kit (Beyotime, Inc.). Image J Analysis Software (USA) was used to analyze scanned images.

### qPCR

After homogenizing the tissue in ice-cold tubes, Trizol was used to extract the total RNA (Phygene, Inc.) followed by manufacturer’s instruction. For reverse transcription with 500ug total RNA, the RT reagent Kit (Biosharp, China) was used to transfer RNA into DNA which will be named as template complementary DNA in the following steps. The CFX96TM real-time PCR detection equipment (Bio-Rad, USA) was used to assess the expression of IL-10, MMP-9, miRNA205-5p genes using real-time quantitative polymerase chain reactions (qPCR). The glyceraldehyde-3-phosphate dehydrogenase (GAPDH) gene was quality control for target gene expression levels. As a dye that binds to amplified DNA and causes it to fluorescence during reactions, SYBR Green (Biosharp, China) was utilized. 10 μl SYBR Green supermix (Biosharp, China), 0.8 μl of sense primer and 0.8 μl of anti-sense primer, 1 μl of complementary DNA template which is above, and 7.4 μl DEPC-treated water were used in the 20 μl reaction mixture. The ^2-∆∆^CT method was used to determine the expression of mRNA in relation to GAPDH. Primer sequences are depicted in Table 1.

**Table 1:** The PCR primer sequences

Gene name	Sequences of primers (5′–3′)
IL-10	Upstream: AAGGCAGTGGAGCAGGTGAA Downstream: CCAGCAGACTCAATACACAC
GAPDH	Upstream: GAAGGGCTCATGACCACAGT Downstream: GGATGCAGGGATGATGTTCT
MMP-9	Upstream: CTTCTGGCGTGTGAGTTTCCA Downstream: ACTGCACGGTTGAAGCAAAGA
mmu-miRNA205-5p	Upstream: TCCGATCACAGTGAACCGGT Downstream: GTGCAGGGTCCGAGGT

### Cell suspension solution of tissue

The tissues are first cutted into small pieces and grinding to mush. The cell suspension was then filtered through 200 mesh nylon mesh (Beyotime, Inc.) to remove tissues that had not been adequately digested. The filtered cell suspension was centrifuged at 1000 rcf for 5 min, the supernatant was discarded, and the precipitate was resuspended with PBS (Beyotime, Inc.), centrifuged at 800 g for 5 min before being neutralized with an equal volume of PBS, washed with PBS once, and resuspended with PBS. After the supernatant had been rinsed with PBS and resuspended, the cell solutions were prepared for use by following Trypan blue stain, ROS and NO detection.

### Trypan blue stain

Combine equal volumes of the 100 µL cell suspension and 100 µL 0.4% Trypan Blue solution (Beyotime, Inc). Allow the mixture to incubate for about 3–5 min at room temperature. Transfer 15 µL of the cell-Trypan Blue mixture onto a hemocytometer or counting slide. Observe the cells under a microscope using brightfield settings. Count both blue-stained cells (indicating dead cells) and unstained cells (indicating viable cells).

### ROS evaluation

The cell suspension is collected and washed, then 1 ml working solution of DCFHDA dye (Biosharp, Inc.) is added to the cell solution and incubated at 37° for 30 min before washing. Finally, the cell solutions can be analyzed using a microplate reader equipment to measure the fluorescence intensity at 525 nm wavelength by using 488 nm laser excitation to quantify the level of ROS.

### Nitric oxide activity detection

Following the manufacturer’s instructions, the biochemical approach for nitrate quantification was used to assess the tissue’s nitric oxide (NO) activity (Solarbio, Inc.). In short, By exposing a 1:5 diluted medium to NADPH (100 mM) and nitrate reductase (250 mU/ml) for 30 min at 37 °C, nitrate was transformed into nitrite. Following treatment with l-glutamine dehydrogenase, the samples were combined with an equivalent volume of newly made Griess reagent. At 540 nm, the absorbance was measured to evaluate NO level.

### COMSOL analysis

A flow-solid coupling module in COMSOL software was used to create an idealized vascular channel model with rectangular openings at both ends. A fixed initial blood flow rate was set. Simulated blood flow inflowed from the left side and outflowed to the right side and simulated acupuncture needle was implemented. The pressure of the vessel and the blood flow velocity were the two main observed quantities.

### Tissue section microfilament stain

Collect the tissue sample of peri-wound acustimulation area and immerse it in Optimal Cutting Temperature (OCT) compound (Biosharp, Inc.) in cryomolds and freeze by liquid nitrogen. Store the frozen tissue blocks at − 20 °C until ready for sectioning. Mount the tissue block on the cryostat chuck using additional OCT compound (Biosharp, Inc.). Cut the tissue sections to a thickness of 8 μm using a cryostat microtome (Leica, Inc). Collect the sections and mount them on clean, positively charged microscope slides (Citotest, Inc.). Fix the tissue sections in 4% paraformaldehyde (Beyotime, Inc.) for 20 min at room temperature. Use PBS to wash the sections three times (Beyotime, Inc.) with each time 5 min to remove the fixative. Permeabilize the sections with a detergent solution 0.1% Triton X-100 in PBS (Beyotime, Inc.) for 10 min and then wash 3 times in PBS (Beyotime, Inc.) with each time 5 min. Incubate the sections with a fluorescent microfilament dye (Beyotime, Inc.) for 45 min. Rinse the sections 3 times in PBS (Beyotime, Inc.) with each time 5 min. Counterstain the nuclei with a fluorescent dye Hochest (Beyotime, Inc.) for 5 min. Rinse the sections again with PBS (Beyotime, Inc.) for 3 times with each time 5 min. Observe the stained sections under a confocal microscope (Leica, Inc.). Analyze the distribution and organization of the microfilaments within the tissue.

### Tissue section immunofluorescence staining

Gather tissue samples, place them in cryomolds using Optimal Cutting Temperature (OCT) compounds (Biosharp, Inc.), and then freeze them with liquid nitrogen. Until they are ready to be sectioned, keep the frozen tissue blocks at − 20 °C. Using more OCT compound (Biosharp, Inc.), mount the tissue block on the cryostat chuck. Using a cryostat microtome, cut the tissue sections to a thickness of 8 μm (Leica, Inc). Mount the sections on sterile, positively charged microscope slides after collecting them (Citotest, Inc.). The tissue sections should be fixed in 4% paraformaldehyde (Beyotime, Inc.) at room temperature for 20 min. To get rid of the fixative, wash the parts three times with PBS (Beyotime, Inc.). After 10 min of permeabilization with a 0.1% Triton X-100 detergent solution in PBS (Beyotime, Inc.), wash the sections three times with PBS (Beyotime, Inc.) for five minutes each. Blocked with 5% bovine serum albumin (BSA) (Beyotime, Inc.) in PBS (Beyotime, Inc.) for 30 min at room temperature. The sections were treated with primary antibodies against ISCU (Sangon Biotech, Inc), MFN1, FIS1, GRP75, GRP78, UCP1, and COL1A1 (Beyotime, Inc.) for an entire night at 4 °C (dilution: 1:500). The sections were treated with the proper fluorophore-conjugated secondary antibodies following three PBS washes (dilution: 1:500) (Beyotime, Inc) for one hour in the dark at room temperature. The sections were then washed with PBS 3 times and mounted with Hochest medium (Beyotime, Inc.) to counterstain the nuclei. Rinse the sections again with PBS (Beyotime, Inc.) 3 times each time for 5 min. Observe the stained sections under a confocal microscope (Leica, Inc.). Analyze the distribution area and intensity of proteins in the tissue.

### Statistical analysis

All data were given as mean with standard error means (SEM) or standard deviation (SD). Data were analyzed using between-subjects variables by t-test followed by two comparison variables. Excel datasheets were used to normalize, and GraphPad Prism 9 was used to analysis and visualize data. The statistical significance level was set at *P* < *0.05*.

## Results

### Anti-inflammatory activity and wound healing effect of TEA

**Fig. 1:**
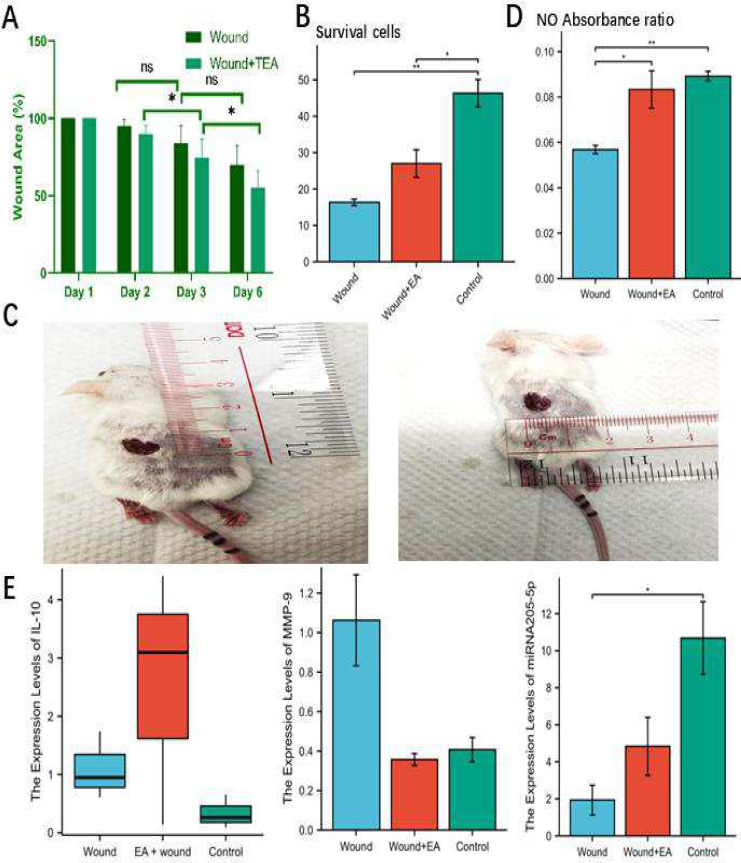
Wound Healing Evaluation, Single Cell Suspension and qPCR on Tissue. **A** Wound area (n = 6); **B** Trypan blue stain of single cell suspensions after skin hole punching (n = 3); **C** On-site wound size measurement; **D** Tissue nitric oxide analysis; **E** qPCR analysis of IL-10, MMP-9, miRNA205-5p genes expression in tissue. N = 3/group, * Indicates significant differences. *p < *0.05*, **p < *0.01*, ***p < *0.001*

**Fig. 2:**
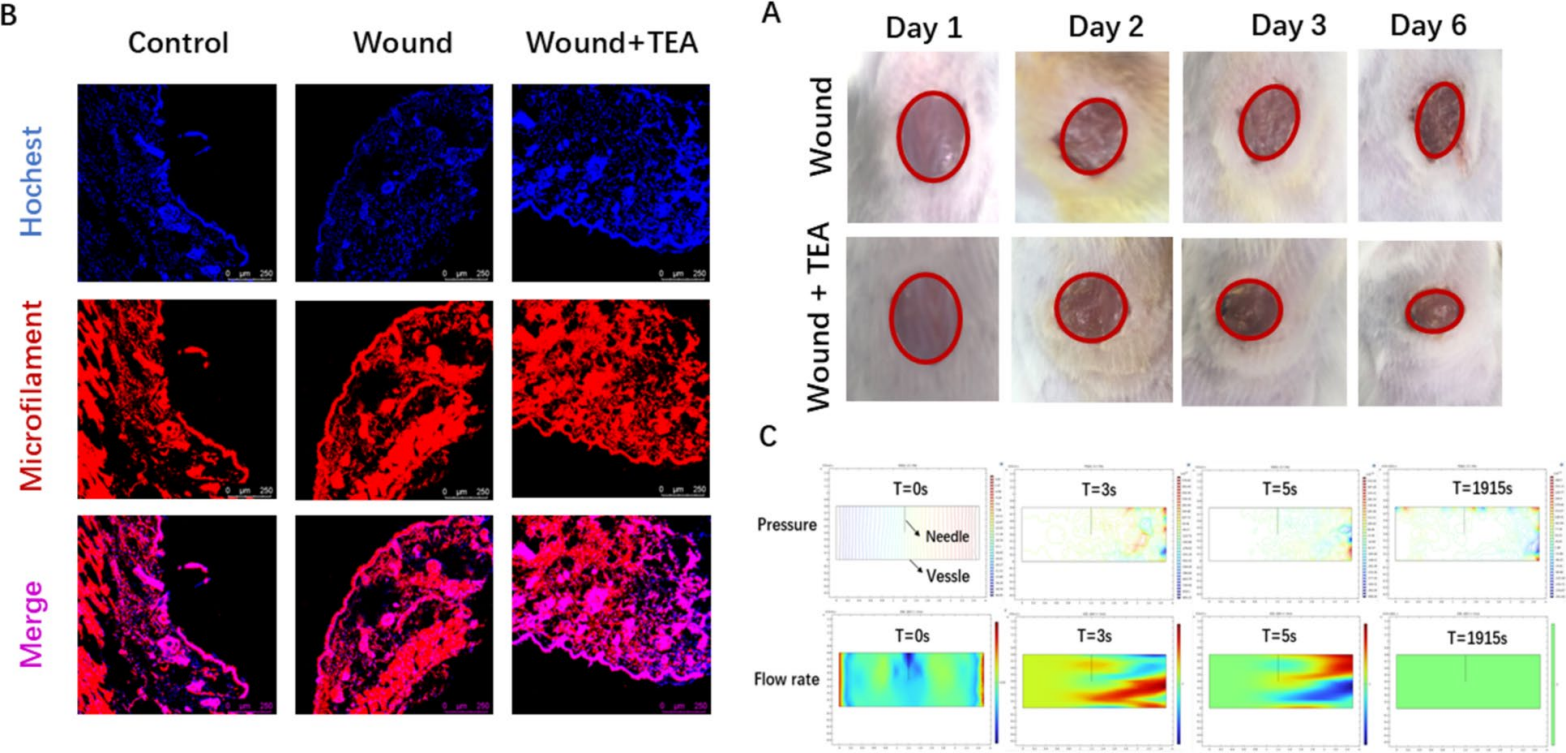
Histological Analysis of Wound Healing and COMSOL Analysis of Vessels. **A** wound area contraction comparison between wound model group and TEA group. **B** microfilament fluorescence staining of peri-wound acupoint skin tissue section. **C** COMSOL analysis of vessels

The wound area of Wound + TEA group is smaller than that of the Wound group through calculating long* short diameter product by use of calipers, which is shown in Fig. 1A. Compared to cell survival in the normal group, cell survival in the wound group was significantly reduced, and after TEA treatment, the wound reduced less cell death (Fig. 1B). Figure 1C shows the procedure of measuring wound area on a mouse wound. Nitric oxide was significantly reduced in the wound group compared to NO in the normal group and after TEA treatment, NO was significantly increased compared to the wound model group (Fig. 1D). qPCR investigations of gene expression levels are depicted in Fig. 1E. IL-10 increased in both wound and wound TEA groups compared to the normal group, but it increased more in the TEA group (Fig. 1E). The Wound + TEA group had increased IL-10 in qPCR analysis compared with the Wound group, indicating an anti-inflammation impact in wound healing. Compared to the normal group, wound group had MMP9 rose, and compared to the wound group, wound TEA group decreased, indicating lower wound inflammation (Fig. 1E). Compared to the normal group, miRNA205-5P was significantly decreased in the wound group and increased after TEA treatment (Fig. 1E), which is targeting PI3K signaling inhibitors [29]. Compared to the wound group, the wound area was smaller after TEA treatment, especially on day 6 (Fig. 2A). Microfilament staining revealed that the current of treatment in the TEA group did not significantly cause tissue damage at the acupuncture points near the wound, compared with wound group that do not have current intervention on acupoints (Fig. 2B). The microfilament skeleton of the peri-wound acupoint skin are a without TEA and the area under TEA, are both intact, suggesting that TEA device is activating the cytokines without causing significant damage to the peri-wound acupoints. By COMSOL analysis, blood flow rate produces a stratified change after manual acupuncture and produces a point-like distribution of pressure along microvasculature (Fig. 2C). Acupuncture needle insertion induced a transient decrease and then a sustained increase in blood flow rate along with a centralized distribution and downward transmission of pressure in the vessel wall. We previously found that TEA stimulates NO, a molecule that stimulates blood microvasculature [4, 31], suggesting vascular signaling involvement. Therefore, manual acupuncture and TEA results are similar in that they both cause vascular stimulation.

### Signaling molecules modulation of TEA signaling

**Fig. 3:**
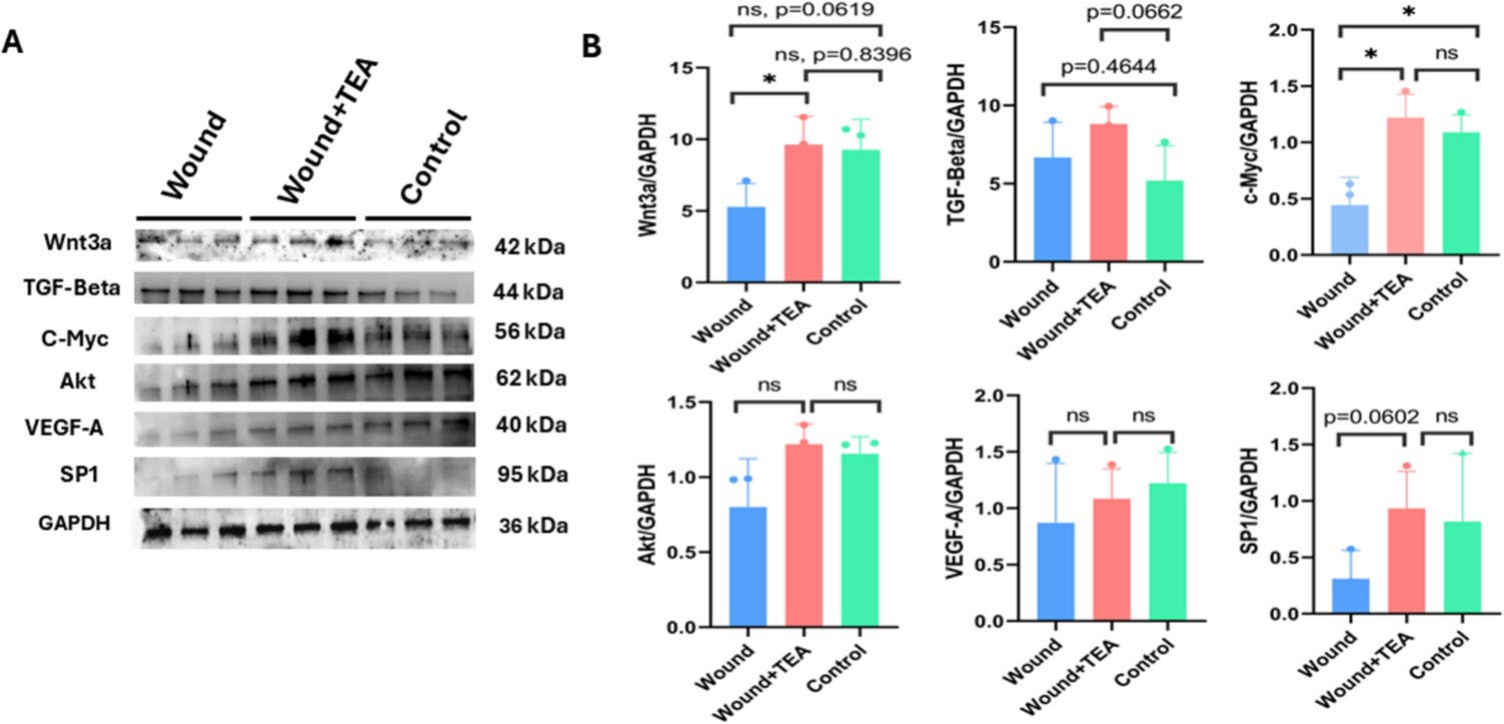
Signaling Molecules Evaluation of Wound Healing. **A** Western blotting of signaling proteins after 7 days; **B** Grey shade analysis of proteins in A. n = 3 for each group, *Indicates significant differences. *p < *0.05*, **p < *0.01*, ***p < *0.001*

Compared to the Control and Wound groups, Fig. 3A depicts western blotting of signaling proteins, indicating electrical acustimulation helps to promote VEGF-A, Akt, c-Myc, SP1, Wnt3a, TGF-Beta expressions in different degrees. Compared to the normal group, the wnt3a expression level was decreased in the wound group, and compared to the wound group, the wnt3a expression level was significantly increased after TEA treatment (*P* < 0.05). Compared to the normal group, TGF-BETA increased in the wound model group, which suggests that the wound model group was also in the process of healing, but TGF-BETA increased more in the wound plus TEA group, which suggests that more wound healing-promoting factor were released from TEA. Compared to the normal group, c-myc was significantly reduced and after TEA treatment, c-myc was significantly increased. Compared to the normal group, Akt was reduced in the wound group, but the level of Akt was elevated after TEA. VEGF-A was reduced in the wound group compared to the normal group, and the level of VEGF-A increased after TEA treatment. Compared to the normal group, SP1 decreased in the wound group and increased after TEA treatment. As shown in Fig. 3B, there is grey shade analysis of proteins from Fig. 3A.

### Recovery after TEA therapy through mitochondrial dynamics, metabolism, and chaperon protein-induced stress response

**Fig. 4:**
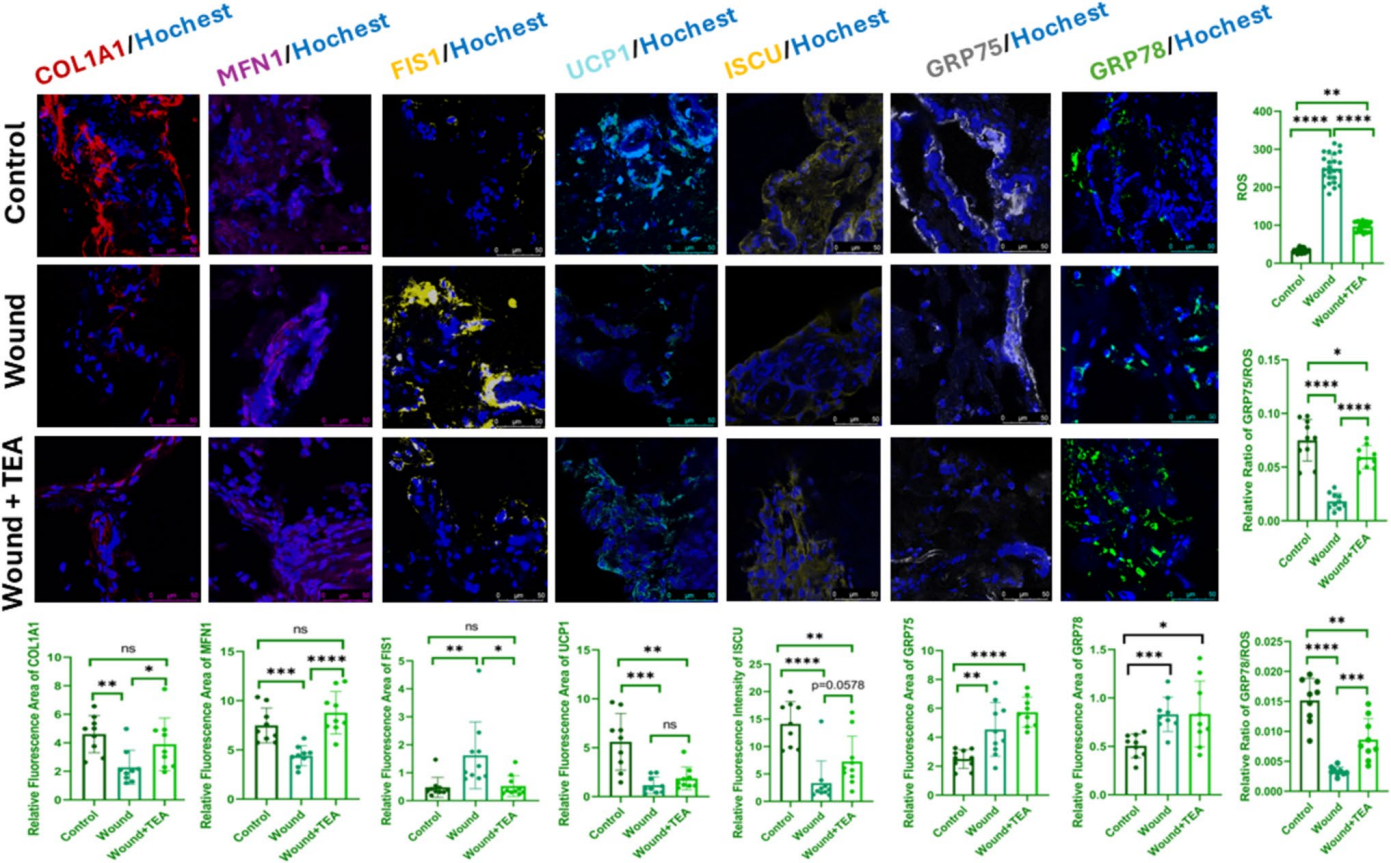
Effects of TEA treatment on mitochondria dynamics, metabolism and chaperon proteins in wound healing. TEA decreased FIS1, and ROS, and increased COL1A1, MFN1, ISCU, UCP1, GRP75, GRP78, and GRP75/ROS, GRP78/ROS. Data are presented as mean ± SEM. *p < 0.05, **p < 0.01, ***p < 0.001, ****p<0.0001

COL1A1 was significantly reduced in the wound group compared to the normal group, while after TEA treatment, COL1A1 levels were significantly increased. Compared to the normal group, MFN1 was significantly reduced and FIS1 was significantly increased, suggesting increased mitochondrial fragmentation. Compared to the wound group, TEA treatment significantly reduced excess FIS1 and significantly increased MFN1 levels, suggesting attenuated mitochondrial fragmentation. Compared to the normal group, UCP1 was significantly reduced in the wound group, suggesting attenuated thermogenic metabolism. After TEA treatment, UCP1 increased. ISCU was significantly reduced in the wound group compared to the normal group and increased after TEA treatment (*P* = 0.0578). Compared to normal group, GRP75, GRP78 were differentially elevated suggesting elevation of molecular chaperone proteins and protective factors. Compared with the normal group, ROS was significantly elevated in the wound group and significantly reduced after TEA treatment. GRP75/ROS, GRP78/ROS were significantly decreased in wound group compared to normal group and significantly increased after TEA treatment (Fig. 4).

## Discussion

Nitric oxide enhances VEGF-A and TGF-β expression and promotes angiogenesis and collagen deposition [30]. SP1 is activated by growth factors such as TGF-β and promotes the expression of genes related to cell growth and migration [31]. When Sp1 is hyperactivated during the healing of epidermal wounds, collagen synthesis may noticeably increase [32]. Akt signaling is activated by VEGF to increase cell survival and proliferation [33]. High concentrations of ROS, on the other hand, may inhibit NO production and cause oxidative damage to the cells, thereby affecting healing [34]. Activation of c-Myc promotes hair growth, and overexpression of Wnt ligands increases the number of regenerating hair follicles [35, 36]. c-Myc could control mouse hair follicle cell cycle and promote follicle proliferation [37]. Wnt signaling pathway involved in wound healing by targeting c-Myc [38]. So, Wnt could target c-Myc to promote wound healing and likely to initiate hair follicles regeneration [38]. MiR-205 genetic deletion results in significant impairment of epidermal and hair follicle growth and newborn mortality [29]. Targeting a negative regulator of PI3K signaling, miR-205 causes the PI3K pathway’s negative regulator to become prematurely raised, which lowers pAkt levels [29], and these mechanisms may have similar biological significance in wound healing. IL-10 uses a STAT3-dependent method to raise VEGF expression [39]. Moreover, IL-10 increases the synthesis of TIMP-1 in human monocytes and suppresses the expression of MMP-9 [40]. Akt activation inhibits excess ROS production in response to oxidative stress [51]. Under conditions of oxidative stress, mitochondrial fragmentation is mainly caused by an increase in fission rather than a decrease in fusion [52].

**Fig. 5:**
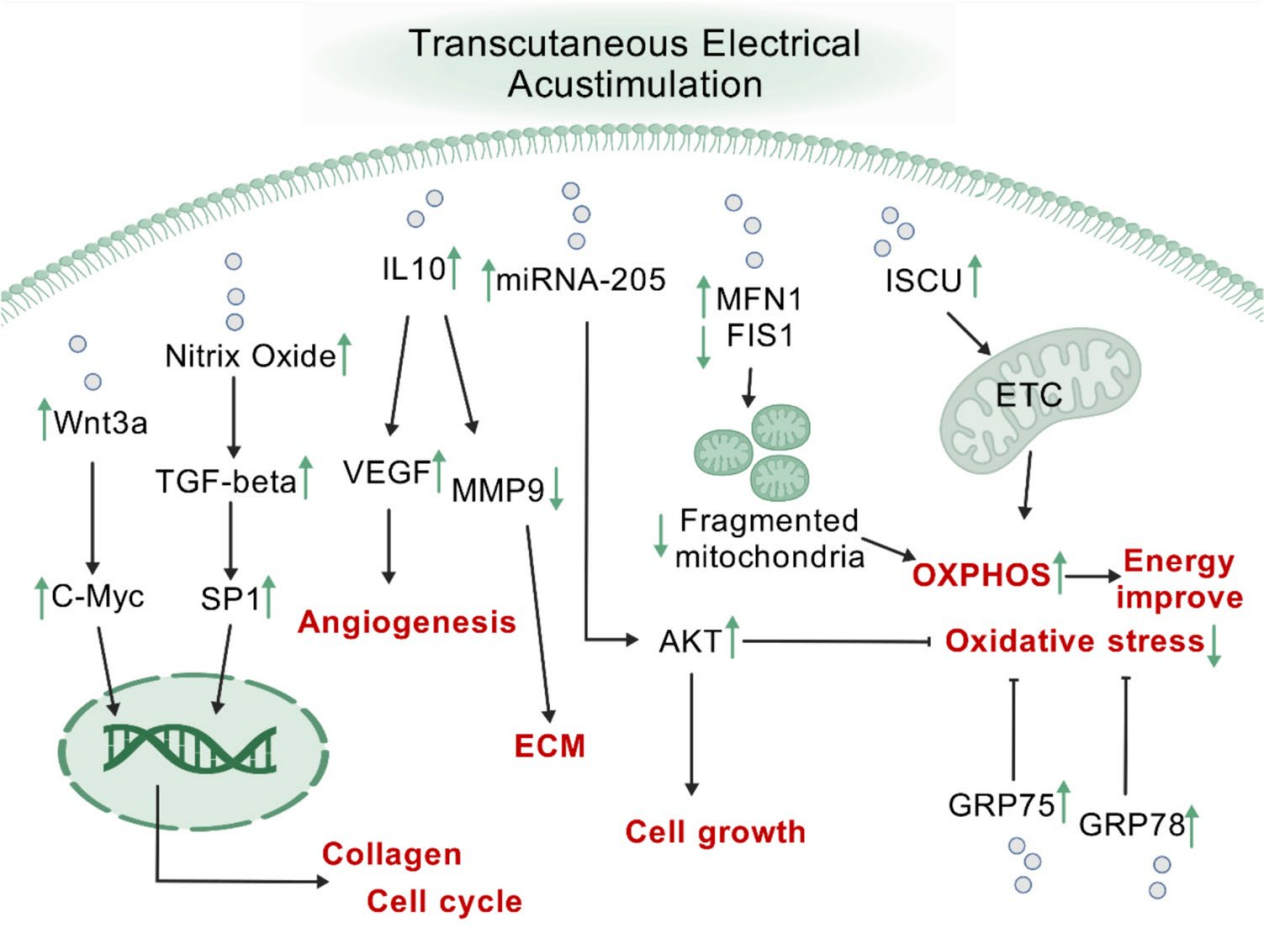
Molecular mechanism of TEA-induced wound healing (Created with BioGDP.com [48])

Some research has shown that wound-induced mitochondrial fragmentation speeds up the closure of epidermal wounds by means of oxidative signaling [41]. However, this was in the early stages of wound model [41, 42]. During the early stages of wound healing, mainly the hemostatic and inflammatory phases, glycolytic metabolism is up-regulated while oxidative metabolism is down-regulated [41, 42]. Later, during the proliferation and remodeling phases, glycolytic metabolism needs to shift to oxidative metabolism to meet energy need in wound healing [42]. During cell division, mitochondrial fusion promotes elevated oxidative phosphorylation [43]. Defects in ISCU or NFS1 disrupt the synthesis of iron-sulfur clusters, leading to a decrease in the activity of mitochondrial complexes that depend on these clusters for electron transfer [44]. The cytoprotective impact of GRP75 overexpression following glucose restriction may be linked to GRP75’s ability to decrease ROS overload [45]. GRP78’s protective action is mediated through the Nrf2/HO-1 signaling pathway [46]. Nrf2 triggers the production of HO-1 under oxidative stress, and HO1 uses heme catabolic byproducts to shield cells from oxidative stress [46]. Iron-sulfur clusters are assembled by ISCU, and since the electron transport chain (ETC) uses a lot of Fe-S clusters, the produced iron-sulfur clusters can be used as feedstock [47]. In light of the findings of earlier research [30–48], we hypothesized that TEA would promote wound healing through a variety of mechanisms (Fig. 5). For other different diseases, some researchers have used TEA parameters of 25 Hz, 10 mA, 1 h [49]. Others have used a stimulation pulse frequency of 25 Hz for 30 min, for two weeks [50]. Although previous electroacupuncture used a current of 1 mA, its duration is 40 min in rats [26]. Our study of mice is shorter in duration and non-invasive, assuming that the skin resistance is calculated to be a minimum of 800 ohms, our applied voltage of 3.7 V will produce a microcurrent of approximately 4.625 mA. Any instrument has requirements for its operating environment. Although we used a 3.7 V non-invasive electro-acupuncture pen instrument, the effects of the laboratory environment needed to be taken into account, and the currents to which the mice were subjected were likely to be at the micro- or milliampere level. An electro-acupuncture device can promote wound healing [26], while an electro-acupuncture pen can achieve the similar effect as an electro-acupuncture device. However, the latter method was to minimize the potential for infection associated with traditional needling, as well as to enhance generalizability to needle-phobic populations.

We have demonstrated that transcutaneous electrical acustimulation (TEA) promotes wound healing in mice by influencing several key RNA and protein molecules, including Wnt3a, SP1, VEGF-A, c-Myc, and TGF-β. These factors play crucial roles in various signaling pathways and regulate mitochondrial dynamics, suggesting parallel relationships among them. However, this study has several limitations. While our findings provide valuable insights into the signaling pathways and cellular processes involved in TEA-mediated wound healing, a more comprehensive analysis of the temporal dynamics and potential crosstalk between these pathways is warranted to elucidate the complex mechanisms underlying these effects. Future research incorporating specific pathway inhibitors or activators could clarify the relative contributions of each signaling cascade to the overall response to TEA. Additionally, including experimental groups with pharmacological manipulation of key pathway components (e.g., Wnt agonists/antagonists) could offer more definitive evidence regarding the specific signaling mechanisms involved. This would enhance our mechanistic understanding and potentially identify novel therapeutic targets for improving wound healing.

As a non-invasive tool derived from traditional acupuncture, TEA is expected to be accessible to a broader population in the future. Given that TEA needs to be administered daily, we speculate that its effects are likely transient, lasting from a few minutes to an hour. We performed electroacupuncture on the final day before tissue collection to study the immediate effects of TEA. Notably, we observed an elevation of GRP75 and GRP78, indicating that TEA may provoke a self-protective response in the body. It is important to note that tissue collection occurred immediately after TEA; thus, the elevation of GRP75 was only transient following TEA treatment. We developed a mathematical model suggesting that GRP75 levels may rise and subsequently decline rather than remain elevated continuously. Importantly, TEA did not induce significant tissue damage, indicating that its therapeutic purpose may be to elicit a more self-protective response without causing harm.

Considering ethical principles in animal research, we aimed to achieve statistically significant results using the minimum number of mice necessary; thus, we utilized only 18 mice to avoid unnecessary use. Our results indicate several molecular changes; however, fully understanding all underlying mechanisms remains complex and necessitates further investigation. While this study presents significant findings, it is important to acknowledge certain limitations related to wound area measurements, imaging clarity, and potential biases in data analysis. Addressing these factors in future research will enhance the robustness of our conclusions and further validate the therapeutic efficacy of TEA in wound healing. The relatively small sample size is another constraint. Future studies should involve larger cohorts. This research represents a preliminary investigation at a specific time point in the wound healing process, highlighting the need for deeper exploration of this biological phenomenon moving forward.

In conclusion, the current study illustrates that transcutaneous electrical acustimulation (TEA) effectively promotes wound healing in mice by modulating the expression of proteins associated with various signaling pathways. These findings contribute to the growing body of evidence supporting TEA as a promising therapeutic strategy for wound management. Further research into the specific signaling mechanisms and their temporal dynamics will enhance our understanding of this intricate biological process.

## Conclusions

In summary, transcutaneous electrical acustimulation (TEA) in mice modulates various signaling molecules, promoting cellular recovery and enhancing mitochondrial function to facilitate the wound healing process.

## Data Availability

All data reported in this paper will be shared by the corresponding author upon request. Any additional information required to reanalyze the data reported in this paper is available from the corresponding author upon request.

## Abbreviations

TEA: Transcutaneous electrical acustimulation
Wnt: Wingless-related integration site
TGF-beta: Transforming growth factor beta
Akt: Protein kinase B (PKB), also known as Akt
c-Myc: Cellular Myelocytomatosis
VEGF-A: Vascular endothelial growth factor A
SP1: Specificity protein 1
NO: Nitric oxide
IL-10: Interleukin 10
miRNA: MicroRNA
MMP-9: Matrix metalloproteinase 9
ROS: Reactive oxygen species
GRP75: Glucose-regulated protein 75
GRP78: Glucose-regulated protein 78
ISCU: Iron-sulfur cluster assembly protein
MFN1: Mitofusin 1
FIS1: Mitochondrial fission 1
WB: Western blotting
QPCR: Quantitative polymerase chain reaction
EMT: Epithelial-to-mesenchymal transition
ECM: Extracellular matrix
OXPHOS: Oxidative phosphorylation
ETC: Electron transport chain
UCP1: Uncoupling protein 1

## References

[1] Rodrigues M, Kosaric N, Bonham CA et al (2019) Wound healing: a cellular perspective. Physiol Rev 99(1):665–706 30475656 10.1152/physrev.00067.2017 PMC6442927

[2] Shi A, Li J, Qiu X et al (2021) TGF-β loaded exosome enhances ischemic wound healing in vitro and in vivo. Theranostics 11(13):6616–6631 33995680 10.7150/thno.57701 PMC8120220

[3] Zhang DL, Gu LJ, Liu L et al (2009) Effect of Wnt signaling pathway on wound healing. Biochem Biophys Res Commun 378(2):149–151 19013436 10.1016/j.bbrc.2008.11.011

[4] Ashmore T, Roberts LD, Morash AJ et al (2015) Nitrate enhances skeletal muscle fatty acid oxidation via a nitric oxide-cGMP-PPAR-mediated mechanism. BMC Biol 13(1):1–17 26694920 10.1186/s12915-015-0221-6 PMC4688964

[5] Teng Y, Fan Y, Ma J et al (2021) The PI3K/Akt pathway: emerging roles in skin homeostasis and a group of non-malignant skin disorders. Cells 10(5):1219 34067630 10.3390/cells10051219 PMC8156939

[6] Gandarillas A, Watt FM (1997) c-Myc promotes differentiation of human epidermal stem cells. Genes Dev 11(21):2869–2882. 10.1101/gad.11.21.2869 9353256 10.1101/gad.11.21.2869 PMC316650

[7] Roy S, Khanna S, Sen CK (2008) Redox regulation of the VEGF signaling path and tissue vascularization: hydrogen peroxide, the common link between physical exercise and cutaneous wound healing. Free Radical Biol Med 44(2):180–192. 10.1016/j.freeradbiomed.2007.01.025 18191754 10.1016/j.freeradbiomed.2007.01.025

[8] Pore N, Liu S, Shu HK, Li B, Haas-Kogan D, Stokoe D, Milanini-Mongiat J, Pages G, O’Rourke DM, Bernhard E, Maity A (2004) Sp1 is involved in Akt-mediated induction of VEGF expression through an HIF-1-independent mechanism. Mol Biol Cell 15(11):4841–4853. 10.1091/mbc.e04-05-0374 15342781 10.1091/mbc.E04-05-0374 PMC524732

[9] Ayavoo T, Murugesan K, Gnanasekaran A (2021) Roles and mechanisms of stem cell in wound healing. Stem Cell Investig 2(8):4. 10.21037/sci-2020-027. (**PMID:33829056;PMCID:PMC8022285**) 10.21037/sci-2020-027 PMC8022285 33829056

[10] Xu XM, Møller SG (2011) Iron–sulfur clusters: biogenesis, molecular mechanisms, and their functional significance. Antioxid Redox Signal 15(1):271–307 20812788 10.1089/ars.2010.3259

[11] Mansy SS, Cowan JA (2004) Iron− sulfur cluster biosynthesis: toward an understanding of cellular machinery and molecular mechanism. Acc Chem Res 37(9):719–725 15379587 10.1021/ar0301781

[12] Read AD, Bentley RE, Archer SL, Dunham-Snary KJ (2021) Mitochondrial iron-sulfur clusters: Structure, function, and an emerging role in vascular biology. Redox Biol 47:102164. 10.1016/j.redox.2021.102164. (**Epub 2021 Oct 12. PMID: 34656823; PMCID: PMC8577454**) 34656823 10.1016/j.redox.2021.102164 PMC8577454

[13] Demling RH (2009) Nutrition, anabolism, and the wound healing process: an overview. Eplasty 9:e9 19274069 PMC2642618

[14] Chen H, Chan DC (2005) Emerging functions of mammalian mitochondrial fusion and fission. Human Mol Genet 14(suppl_2):R283–R289 16244327 10.1093/hmg/ddi270

[15] Szabo A, Sumegi K, Fekete K, Hocsak E, Debreceni B, Setalo G Jr, Sumegi B (2018) Activation of mitochondrial fusion provides a new treatment for mitochondria-related diseases. Biochem Pharmacol 150:86–96 29378182 10.1016/j.bcp.2018.01.038

[16] Bond LM, Ntambi JM (2018) UCP1 deficiency increases adipose tissue monounsaturated fatty acid synthesis and trafficking to the liver. J Lipid Res 59(2):224–236. 10.1194/jlr.M078469. (**Epub 2017 Dec 3. PMID: 29203476; PMCID: PMC5794418**) 29203476 10.1194/jlr.M078469 PMC5794418

[17] Wang G, Fan Y, Cao P, Tan K (2022) Insight into the mitochondrial unfolded protein response and cancer: opportunities and challenges. Cell Biosci 12(1):18 35180892 10.1186/s13578-022-00747-0 PMC8857832

[18] Wang N, Ma J, Ma Y, Lu L, Ma C, Qin P, Gao E, Zuo M, Yang J, Yang L (2021) Electroacupuncture pretreatment mitigates myocardial ischemia/reperfusion injury via XBP1/GRP78/Akt pathway. Front Cardiovasc Med 14(8):629547. 10.3389/fcvm.2021.629547 10.3389/fcvm.2021.629547 PMC8236521 34195232

[19] Li Y, Xu G, Hu S et al (2021) Electroacupuncture alleviates intestinal inflammation and barrier dysfunction by activating dopamine in a rat model of intestinal ischemia. Acupunct Med 39(3):208–216 32517478 10.1177/0964528420922232

[20] Hu S, Stern RM, Koch KL (1992) Electrical acustimulation relieves vection-induced motion sickness. Gastroenterology 102(6):1854–1858 1587405 10.1016/0016-5085(92)90305-i

[21] Kim SB, Kim JY, Park SW, Lee NR, Lee SW, Kim YH, Lee YH (2012) Comparison of 2 methods of non-invasive treatment between transcutaneous electrical stimulation and pulsed electromagnetic field stimulation as replacement of invasive manual acupuncture. Acupunct Electrother Res 37(4):247–261. 10.3727/036012912x13831831256294 23409610 10.3727/036012912x13831831256294

[22] Alam MJ, Chen JD (2023) Non-invasive neuromodulation: an emerging intervention for visceral pain in gastrointestinal disorders. Bioelectronic Medicine 9(1):27 37990288 10.1186/s42234-023-00130-5 PMC10664460

[23] Thakral G, Lafontaine J, Najafi B, Talal TK, Kim P, Lavery LA (2013) Electrical stimulation to accelerate wound healing. Diabet Foot Ankle 16:4. 10.3402/dfa.v4i0.22081.PMID:24049559;PMCID:PMC3776323 10.3402/dfa.v4i0.22081 PMC3776323 24049559

[24] Rajendran SB, Challen K, Wright KL, Hardy JG (2021) Electrical stimulation to enhance wound healing. J Funct Biomater 12(2):40. 10.3390/jfb12020040.PMID:34205317;PMCID:PMC8293212 34205317 10.3390/jfb12020040 PMC8293212

[25] Long Y, Wei H, Li J, Yao G, Yu B, Ni D, Wang X (2018) Effective wound healing enabled by discrete alternative electric fields from wearable nanogenerators. ACS Nano 12(12):12533–12540 30488695 10.1021/acsnano.8b07038 PMC6307171

[26] Du W, Bao G, Hu H et al (2020) An mRNA sequencing analysis of the healing-promoting role of electroacupuncture in rat skin wound model. Ann Palliat Med 9:1462–1475 32692201 10.21037/apm-20-626

[27] Du W, Wang Z, Dong Y, Hu H, Zhou H, He X, Hu J, Li Y (2023) Electroacupuncture promotes skin wound repair by improving lipid metabolism and inhibiting ferroptosis. J Cell Mol Med 27(16):2308–2320. 10.1111/jcmm.17811 37307402 10.1111/jcmm.17811 PMC10424292

[28] Du W, He L, Wang Z, Dong Y, He X, Hu J, Zhang M (2023) Serum lipidomics-based study of electroacupuncture for skin wound repair in rats. J Cell Mol Med 27(20):3127–3146 37517065 10.1111/jcmm.17891 PMC10568671

[29] Wang D, Zhang Z, O’Loughlin E, Wang L, Fan X, Lai EC, Yi R (2013) MicroRNA-205 controls neonatal expansion of skin stem cells by modulating the PI(3)K pathway. Nat Cell Biol 15(10):1153–1163. 10.1038/ncb2827 23974039 10.1038/ncb2827 PMC3789848

[30] Sivaraj D, Noishiki C, Kosaric N, Kiwanuka H, Kussie HC, Henn D, Gurtner GC (2023) Nitric oxide-releasing gel accelerates healing in a diabetic murine splinted excisional wound model. Front Med 10:1060758 10.3389/fmed.2023.1060758 PMC10045479 36999070

[31] Ding A, Bian YY, Zhang ZH (2020) SP1/TGF-β1/SMAD2 pathway is involved in angiogenesis during osteogenesis. Mol Med Rep 21(3):1581–1589 32016481 10.3892/mmr.2020.10965 PMC7003058

[32] Nirodi CS, Devalaraja R, Nanney LB, Arrindell S, Russell S, Trupin J, Richmond A (2000) Chemokine and chemokine receptor expression in keloid and normal fibroblasts. Wound Repair Regen 8:371–382 11115149 10.1111/j.1524-475x.2000.00371.x PMC3140346

[33] Cui MD, Pan ZH, Pan LQ (2017) Danggui buxue extract-loaded liposomes in thermosensitive gel enhance in vivo dermal wound healing via activation of the VEGF/PI3K/Akt and TGF-β/Smads signaling pathway. Evid-Based Complement Alternat Med 2017(1):8407249 29292400 10.1155/2017/8407249 PMC5674729

[34] Khorsandi K, Hosseinzadeh R, Esfahani H, Zandsalimi K, Shahidi FK, Abrahamse H (2022) Accelerating skin regeneration and wound healing by controlled ROS from photodynamic treatment. Inflamm Regen 42(1):40. 10.1186/s41232-022-00226-6.PMID:36192814;PMCID:PMC9529607 36192814 10.1186/s41232-022-00226-6 PMC9529607

[35] Ito M, Yang Z, Andl T, Cui C, Kim N, Millar SE et al (2007) Wnt-dependent de novo hair follicle regeneration in adult mouse skin after wounding. Nature 447:316–320. 10.1038/nature05766 17507982 10.1038/nature05766

[36] Wang N, Yang T, Li J, Lei M, Shi J, Qiu W et al (2012) The expression and role of c-Myc in mouse hair follicle morphogenesis and cycling. Acta Histochem 114:199–206. 10.1016/j.acthis.2011.04.009 21621827 10.1016/j.acthis.2011.04.009

[37] Wang N, Yang T, Li J, Lei M, Shi J, Qiu W, Lian X (2012) The expression and role of c-Myc in mouse hair follicle morphogenesis and cycling. Acta Histochem 114(3):199–206 21621827 10.1016/j.acthis.2011.04.009

[38] Shi Y, Shu B, Yang R, Xu Y, Xing B, Liu J, Chen L, Qi S, Liu X, Wang P, Tang J, Xie J (2015) Wnt and Notch signaling pathway involved in wound healing by targeting c-Myc and Hes1 separately. Stem Cell Res Ther 6(1):120. 10.1186/s13287-015-0103-4 26076648 10.1186/s13287-015-0103-4 PMC4501079

[39] Short WD, Steen E, Kaul A, Wang X, Olutoye OO, Vangapandu HV, Balaji S (2022) IL-10 promotes endothelial progenitor cell infiltration and wound healing via STAT3. FASEB J. 10.1096/fj.201901024RR 35670763 10.1096/fj.201901024RR PMC9796147

[40] Lacraz S, Nicod LP, Chicheportiche R, Welgus HG, Dayer JM (1995) IL-10 inhibits metalloproteinase and stimulates TIMP-1 production in human mononuclear phagocytes. J Clin Invest 96(5):2304–2310. 10.1172/JCI118286 7593617 10.1172/JCI118286 PMC185881

[41] Fu H, Zhou H, Yu X, Xu J, Zhou J, Meng X, Xu S (2020) Wounding triggers MIRO-1 dependent mitochondrial fragmentation that accelerates epidermal wound closure through oxidative signaling. Nat Commun 11(1):1050 32103012 10.1038/s41467-020-14885-x PMC7044169

[42] Hunt M, Torres M, Bachar-Wikström E, Wikström JD (2023) Multifaceted roles of mitochondria in wound healing and chronic wound pathogenesis. Front Cell Dev Biol 11:1252318 37771375 10.3389/fcell.2023.1252318 PMC10523588

[43] Yao CH, Wang R, Wang Y, Kung CP, Weber JD, Patti GJ (2019) Mitochondrial fusion supports increased oxidative phosphorylation during cell proliferation. Elife 8:e41351 30694178 10.7554/eLife.41351 PMC6351101

[44] Vanlander AV, Van Coster R (2018) Clinical and genetic aspects of defects in the mitochondrial iron–sulfur cluster synthesis pathway. J Biol Inorg Chem 23:495–506 29623423 10.1007/s00775-018-1550-z PMC6006192

[45] Liu Y, Liu W, Song XD, Zuo J (2005) Effect of GRP75/mthsp70/PBP74/mortalin overexpression on intracellular ATP level, mitochondrial membrane potential and ROS accumulation following glucose deprivation in PC12 cells. Mol Cell Biochem 268:45–51 15724436 10.1007/s11010-005-2996-1

[46] Ji H, Xiao F, Li S, Wei R, Yu F, Xu J (2021) GRP78 effectively protect hypoxia/reperfusion-induced myocardial apoptosis via promotion of the Nrf2/HO-1 signaling pathway. J Cell Physiol 236(2):1228–1236. 10.1002/jcp.29929 32657424 10.1002/jcp.29929 PMC7754434

[47] Barupala DP, Dzul SP, Riggs-Gelasco PJ, Stemmler TL (2016) Synthesis, delivery and regulation of eukaryotic heme and Fe–S cluster cofactors. Arch Biochem Biophys 592:60–75 26785297 10.1016/j.abb.2016.01.010 PMC4784227

[48] Jiang S, Li H, Zhang L, Mu W, Zhang Y, Chen T, Wu J, Tang H, Zheng S, Liu Y, Wu Y, Luo X, Xie Y, Ren J (2024) Generic diagramming platform (GDP): a comprehensive database of high-quality biomedical graphics. Nucleic Acids Res. 10.1093/nar/gkae973 39470721 10.1093/nar/gkae973 PMC11701665

[49] Huang Z, Lin Z, Lin C, Chu H, Zheng X, Chen B, Du L, Chen JDZ, Dai N (2022) Transcutaneous electrical acustimulation improves irritable Bowel Syndrome with constipation by accelerating colon transit and reducing rectal sensation using autonomic mechanisms. Am J Gastroenterol 117(9):1491–1501. 10.14309/ajg.0000000000001882. (**Epub 2022 Jun 10 PMID: 35973183**) 35973183 10.14309/ajg.0000000000001882

[50] Liu J, Dai Q, Qu T, Ma J, Lv C, Wang H, Yu Y (2024) Ameliorating effects of transcutaneous auricular vagus nerve stimulation on a mouse model of constipation-predominant irritable bowel syndrome. Neurobiol Dis 193:106440 38369213 10.1016/j.nbd.2024.106440

[51] Huang TL, Jiang WJ, Zhou Z, Shi TF, Yu M, Yu M, Li L (2024) Quercetin attenuates cisplatin-induced mitochondrial apoptosis via PI3K/Akt mediated inhibition of oxidative stress in pericytes and improves the blood labyrinth barrier permeability. Chemico-Biol Interact 393:110939 10.1016/j.cbi.2024.110939 38490643

[52] Yu T, Wang L, Zhang L, Deuster PA (2023) Mitochondrial fission as a therapeutic target for metabolic diseases: insights into antioxidant strategies. Antioxidants 12(6):1163 37371893 10.3390/antiox12061163 PMC10295595

